# Association between erythrocyte folate and pregnancy: A scientometric analysis

**DOI:** 10.12669/pjms.41.10.12671

**Published:** 2025-10

**Authors:** Yu Xia, Xuanyi Wang, Datong Liu, Limei Sun, Shen Li, Meiling Sun

**Affiliations:** 1 Yu Xia, Department of Obstetrics, Rizhao People’s Hospital, Rizhao, Shandong Province 276899, P.R. China; 2 Xuanyi Wang, 193 Ferry Rd, Southport, Qld 4215, Australia; 3 Datong Liu, Jining Medical University, Jining City, Shandong Province 272067, P.R. China; 4 Limei Sun, Department of Obstetrics, Rizhao People’s Hospital, Rizhao, Shandong Province 276899, P.R. China; 5 Shen Li, Department of Obstetrics, Rizhao People’s Hospital, Rizhao, Shandong Province 276899, P.R. China; 6 Meiling Sun, Department of Obstetrics, Rizhao People’s Hospital, Rizhao, Shandong Province 276899, P.R. China

**Keywords:** CiteSpace, Erythrocyte folate, Neural tube defects, Pregnancy, Scientometrics, Visual analysis, VOSviewer

## Abstract

**Objective:**

This study aimed to conduct a scientometric analysis of the trends and patterns in studies on the association between erythrocyte folic acid and pregnancy.

**Methodology:**

The core dataset of Web of Science was searched for literatures on the association between erythrocyte folate and pregnancy, published between January 1980 to December 2023. CiteSpace6.1. R6(64-bit) and VOSviewer 1.6.19 software were used to visually analyze the network of keywords, countries, authors, institutions, fields and funds included in the studies.

**Results:**

A total of 243 studies were analyzed. The most widely covered fields and journals are Nutrition & Dietetics and The American Journal of Clinical Nutrition (AJCN), respectively. The most collaborative authors, institutions and countries are: Czeizel AE, Centers for Disease Control and Prevention (CDC), the United States of America (USA), respectively. Key words clustering and emergence analysis identified 13 clusters. The research in the field was mainly reflected in the long- and short-term impact of red blood cells and related Vitamin- B12, homocysteine and methylene tetrahydrofolate reductase on pregnant women and infants. The association between erythrocyte folic acid, neural tube development abnormality and the reduction of maternal and infant complications by folic acid supplementation has been the focus of research in this field.

**Conclusion:**

Erythrocyte folate is undoubtedly associated with pregnancy, with risk factor and prevention may be the focus of future research. With strong publishing rates, the USA and UK have become the main research nations in this field. The key studies found that the prevention of NTDs require increased folic acid intake of 0.4mg/day, but improved dietary guidance to help pregnant women meet but not exceed dietary recommendations is warranted.

## INTRODUCTION

Folic acid, also known as Vitamin-B9 and Vitamin- M, is involved in nucleotide synthesis in vivo, plays a vital role in cell division and proliferation and participates in amino acid metabolism and methylation.^1^ Folic acid deficiency may impact fetal development, leading to fetal neural tube malformations.^2^ Since folic acid cannot be synthesized and stored in the human body, it can only be obtained through the diet.^3^ In normal adults, folic acid intake from food can meet daily needs, while during pregnancy, decreased maternal gastric acid secretion and folic acid absorption rate, coupled with increased renal blood flow and lower renal tubule absorption, result in increased folic acid output.^4^ Since 50-90% of folic acid in food may be lost through cooking and processing,^5^ pregnant women need to ensure the appropriate amount of folic acid through synthetic folic acid supplements, which have high bioavailability and better stability. Current guidelines clearly recommend the supplementation of 0.4mg of folic acid per day for pregnant women to improve maternal and infant outcomes. The effectiveness and acceptance of such recommendations, however, may be influenced by cultural and regional factors, as observed in a study by Nisar et al.^6^ , which explored the perceptions of antenatal supplements in different populations.^7^

Scientometrics, a method for analyzing quantitative patterns in scientific literature, offers valuable insights that extend beyond traditional narrative reviews. Unlike qualitative syntheses, scientometrics enables the analysis of large-scale datasets to identify long-term trends, emerging topics, and key contributors in a field. This approach allows researchers to explore the evolving landscape of scientific knowledge, particularly in folate supplementation during pregnancy.^8^ Using scientometric tools like CiteSpace and VOSviewer, this study visualizes co-occurrence networks of keywords, co-citations, and author collaborations, uncovering dynamic shifts in research focus and highlighting previously overlooked directions. This methodology provides a more comprehensive and objective understanding of research trends, helping to reveal new research themes and the interconnectedness of research clusters across countries and institutions. These insights offer a clearer picture of how folate supplementation research has developed and where it is heading, offering a richer perspective than traditional narrative reviews. The results may provide more reasonable clinical guidance for personalized folic acid supplementation through the detection of red blood cell folic acid levels, improve maternal and infant outcomes and provide scientific suggestions for research.

## METHODOLOGY

Data for this study were collected from the Web of Science Core Collection (WOSCC), a widely used resource for scientometric analysis due to its comprehensive citation and bibliometric data. The scientometric approach employed includes advanced tools such as CiteSpace and VOSviewer , which facilitate the visualization and analysis of research patterns by mapping collaborative networks, keyword co-occurrence, and citation dynamics. Unlike traditional narrative reviews, which primarily offer qualitative summaries, scientometrics allows for the identification of evolving research trends, key contributors, and emerging topics. These tools reveal dynamic shifts in research focus that may not be easily captured through conventional review methods, providing a more comprehensive understanding of the scientific discourse over time.^9^ The search strategy was as follows: (TS= (“erythrocyte folic acid” or “red blood cell folic” or “erythrocyte folate” or “red blood cell folate” or “red cell folate” or “blood cells folic acid”)) AND TS= (“pregnancy” or “pregnant” or “gestation” or “gestational” or “gravidity”).

### The inclusion criteria:

- Studies on the association between erythrocyte folate and pregnancy from January 1980 to December 2023.
- English-language studies.
- The article type limited to “article” or “review”.

### The exclusion criteria:

- Article type such as meeting abstract, letter, editorial material, news item and book review.
- Duplicate data (eliminated with CiteSpace).

### Data analysis:

The software VOSviewer 1.6.19 was used to obtain the co-occurrence keyword network. CiteSpace6.1. R6(64-bit) was used to extract collaborative networks (countries, institutions, funds, authors), co-citation analysis (co-citation authors, co-citation journals, co-citation literature clustering) and co-occurrence analysis (co-occurrence author keywords and literature domain network) and carried out burstness analysis, timeline analysis and draw impact flow diagram. The influence of co-cited literature and various important indicators generated by CiteSpace were analyzed, including temporal indicators such as (burstness analysis), structural indicators such as centrality, modularity (Q score) and silhouette score (S score), Q scores from 0 to +1, S scores from −1 to +1. When Q is greater than 0.3, the clustering structure is considered significant and when S is greater than 0.3, 0.5, or 0.7, the network is considered homogeneous, reasonable, or highly trusted, respectively. Cluster labels were generated from noun phrases in the list of article keywords cited in each cluster using a likelihood ratio test (p < 0.001). According to Price’s law [8], P = 0.749 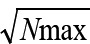 (Nmax is the maximum number of papers published by the author and the number of papers greater than P is the core author)

## RESULTS

### Publication Trend:

**Fig.1:**
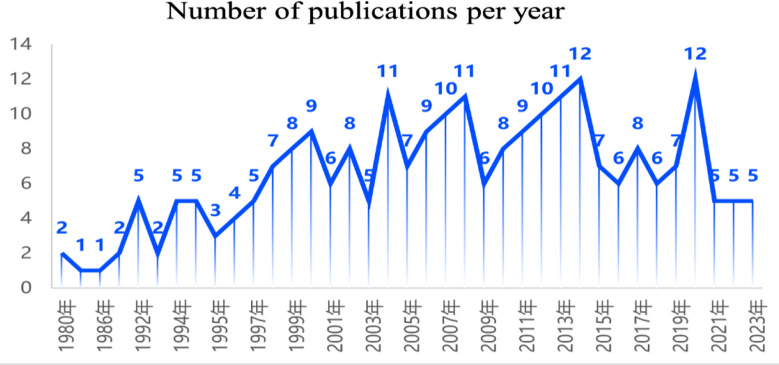
Trend analysis of publications in 1980-2023.

A total of 243 articles were included. The annual number of published papers can represent the change trend of research in a field,^10^ as shown in Fig.1. From 1980 to 2015, the number of published articles rose slowly in a fluctuating manner. The number of published papers decreased from 2015 to 2020, increased again in 2021, and then stabilized at five papers per year, indicating that studies on the association between red blood cell folic acid and pregnancy have received consistent attention. The rise in publications from 2021 onward can be attributed to several factors, including increased global health initiatives reinforcing the importance of folate supplementation for preventing neural tube defects (NTDs), advances in research methodologies such as improved biomarkers for folate status, heightened public health awareness campaigns, and the impact of the COVID-19 pandemic on global maternal health research.

### Distribution of literature research fields and journals:

**Supplementary Fig.1:**
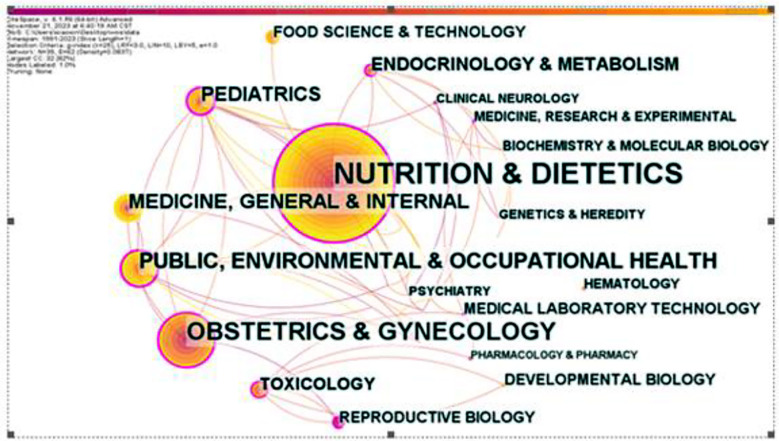
Distribution of research fields.

The top five research fields and volume of literature published were Nutrition and Dietetics (85), Obstetrics and Gynecology (40), Public, Environmental and Occupational Health (27), Medicine General & Internal (21) and Pediatrics (20). (Supplementary Fig.1). The top five journals with the highest total citation frequency and citation frequency were The American Journal of Clinical Nutrition (AJCN) (182), Lancet (163), The Journal of Nutrition (JN) (153), The New England Journal of Medicine (NEJM) (131) and The Journal of the American Medical Association (JAMA) (119). The impact factors (IF) of frequently cited journals were consulted to assess the influence of the study in the field. IF refers to the ratio of the total number of citations of papers published in the previous two years of a journal to the total number of papers published in the same period. Lancet, NEJM and JAMA were identified as frequently cited journals with the highest impact factors, indicating high quality of research in this field (Table-I).

**Table-I:** Top 10 journals with cited frequency.

NO.	Count	Centrality	Year	Cited Journal	IF/Year
1	182	0.01	1991	The American Journal of Clinical Nutrition (AJCN )	7.1/2022
2	163	0.03	1992	LANCET	168.9/2022
3	153	0.03	1991	The Journal of Nutrition (JN)	4.2/2022
4	131	0.01	1993	The New England Journal of Medicine (NEJM)	158.5/2022
5	119	0.02	1992	The Journal of the American Medical Association (JAMA)	120.7/2022
6	81	0.1	1991	American Journal of Obstetrics and Gynecology (AJOG)	9.8/2022
7	80	0.09	1991	The British Journal of Nutrition (BJN)	3.6/2022
8	73	0.09	1991	Clinical Chemistry	9.3/2022
9	67	0.02	1998	The European Journal of Clinical Nutrition (EJCN)	4.7/2022
10	65	0.13	1994	The American Journal of Epidemiology (AJE )	5.0/2022

### Network of institutional and national collaboration:

**Supplementary Fig.2:**
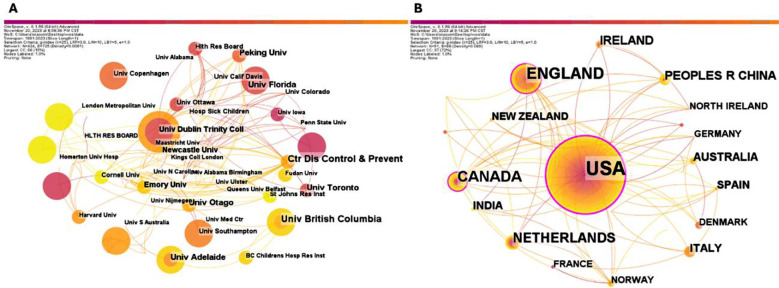
Network of institutional and national collaboration from 1990 to 2023. A) Institutional Collaboration Network; B) National Collaboration Network.

Both institutional and national collaborative networks are working closely on the association between erythrocyte folate and pregnancy, which indicates that the research in this field draws high attention in various countries and institutions. The institutions that publish more papers were: The Centers for Disease Control and Prevention of the United States, British Columbia University (9), the University of Toronto, Peking University and others (6) (Supplementary Fig.2A). The top five countries included the United States with 74 articles, the United Kingdom with 33 articles, Canada with 27 articles, the Netherlands with 20 articles and China and Ireland with 14 articles (Supplementary Fig.2B).

### Author collaboration network:

**Supplementary Fig.3:**
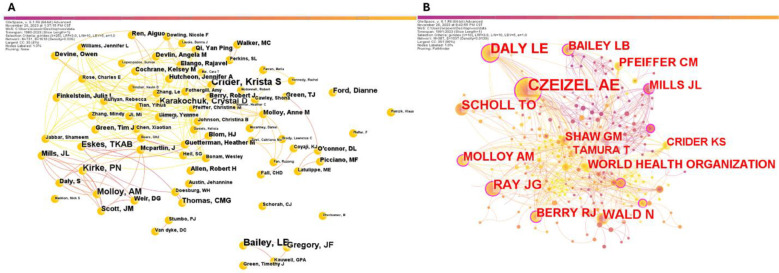
Author collaboration network. A) Core authors; B) Authors’ co-citation network.

Analyzing the author collaboration network allows to roughly understand the collaboration between leading researchers and teams in the field. This study included 731 authors and selected 102 core authors who published more than two articles according to Price’s Law formula. A team collaboration network diagram was formed with Bailey LB, Crider Krista S, Kirke PN, Molloy AM and others as the core authors, as shown in Supplementary Fig.3A. The authors’ co-citation network and top five citation frequency were analyzed as follows: Czizel Ae (86), Daly Le (44), Ray JG (38), Scholl to (33) and Wald N (32). Node size represents the citation frequency, as shown in Supplementary Fig.3B.

### Keyword co-occurrence, clustering and emergent analysis:

### Keyword co-occurrence and cluster analysis:

**Supplementary Fig.4:**
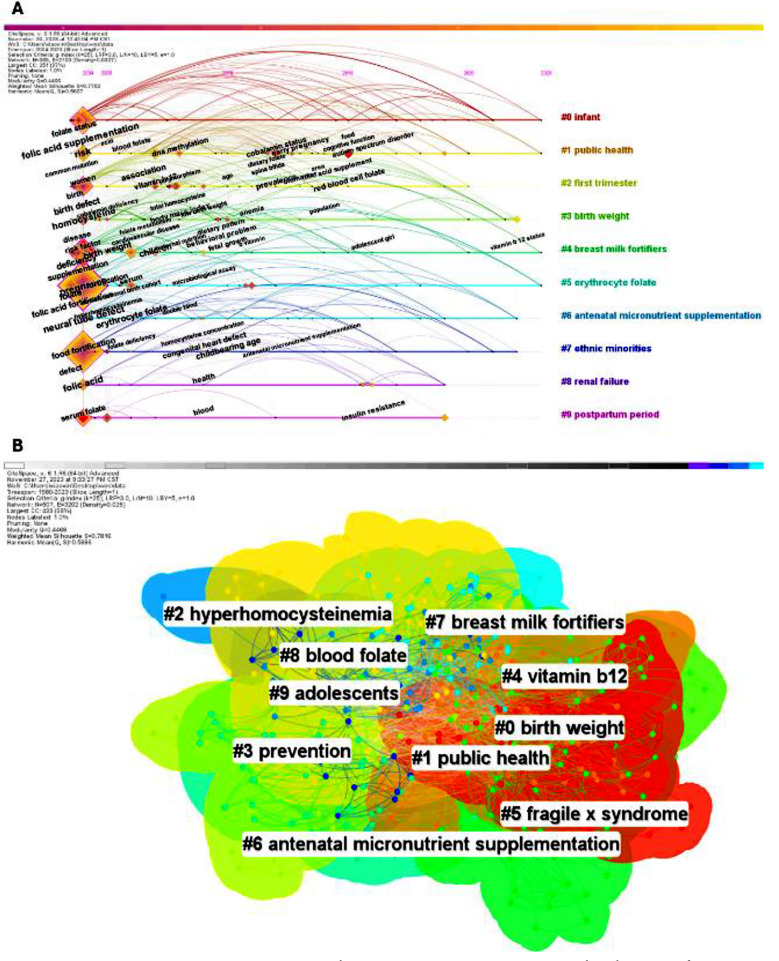
Keyword co-occurrence and clustering analysis. A) Keyword co-occurrence network; B) Clusters of co-occurring keywords networks in 1980-2023.

The hot spots and trends in the research field can be understood through the analysis of keywords. The co-occurrence network of 101 high-frequency keywords with frequency ≥5 obtained from VOSviewer is shown in Supplementary Fig.4A, where nodes represent keywords and larger nodes indicate higher frequency; the lines between the nodes represent the co-occurrence times of keywords; different colors represent different publication times. The top five keywords that appeared more frequently were: “folic acid” + “folic-acid” (120), pregnancy (101), neural tube defects (NTDs) (91), folate (89) and homocysteine (57).

The local linear regression method (LLR) was then used to cluster the keyword network and 13 clusters were obtained (Q=0.4468, S=0.7816), indicating that the cluster structure of this time was significant. As shown in Supplementary Fig.4B, the top 10 displayed clusters included #0 birth weight, #1 public health, # 2 birth weight, # 2 public health, # 2 birth weight, # 3 birth weight, # 3 public health. #2 hyperhomocysteinemia, #3 prevention, #4 Vitamin- b 12, #5 fragile x syndrome, #6 antenatal Vitamin- supplementation, #7 breast milk fortifier, #8 blood folate, #9 adolescents. The relevant information of the 10 clusters is summarized in Table-II. Cluster analysis shows that the research in this field is mainly reflected in the long- and short-term impact of red blood cells and related Vitamin- B12, homocysteine and methylene tetrahydrofolate reductase on pregnant women and infants. The association between clusters was reflected in the close combination of clinical and mechanistic studies, forming a complete research system.

**Table-II:** Top 10 cluster labels of the association between RBC folic acid and pregnancy.

Cluster ID	Cluster name	Size	Silhouette	Mean (year)	Top 5 terms (LSI)
#0	Birth weight	57	0.784	2007	Birth Weight; Gestational Week; Folic Acid; Intra Uterine; Preterm Birth
#1	Public health	53	0.769	2007	Neural tube defects; Vitamin- b-12; Pregnant women; Dietary assessment; Recurrent miscarriage
#2	Hyperhomocysteinemia	51	0.721	2000	Hyperhomocysteinemia; for gestational age; fetal; insulin resistance; glucose
#3	Prevention	51	0.66	2001	NTDS; Steady State; Folic Acid Supplementation; Pregnant Women; Elimination Kinetics
#4	Vitamin- b12	47	0.789	2003	Vitamin- b12; Reference interval; Postpartum period; Erythrocyte indices; Vitamin- b-12
#5	Fragile x syndrome	44	0.814	2011	Periconceptional Vitamin-; Neural Tube; Folic Acid; Birth Weight; Chronic Phenytoin
#6	Antenatal micronutrient supplementation	41	0.796	2009	Folic Acid; Coronary Heart; Cystathionine Beta; 10 Methylenetetrahydrofolate Reductase; Plasma Homocysteine
#7	Breast milk fortifiers	38	0.788	2005	Birth defects; Serum Folate Concentrations; First Trimester; Dietary Folate Intake; Vitamin B12
#8	Blood folate	38	0.755	2007	NTDS; Zinc Absorption; Zinc Status; Whole-Body Counting; Birth Defects
#9	Adolescents	26	0.893	1999	Adolescents; Vitamin B-12; Folate; Lactation; Iron

### Key words time diagram, outburst and impact flow diagram analysis:

**Supplementary Fig.5:**
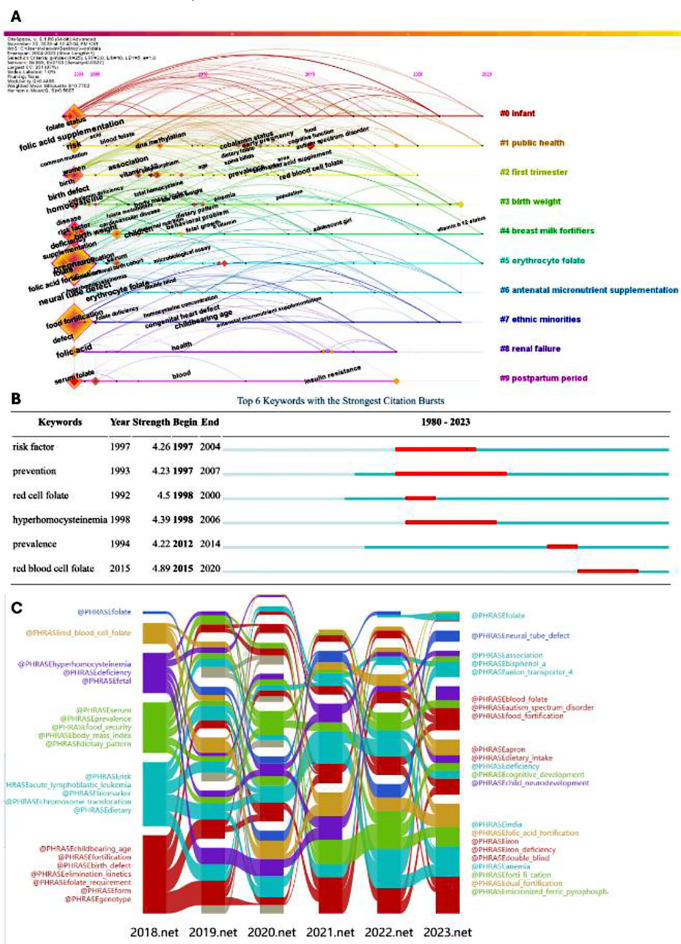
Keywords time diagram, outburst and impact flow diagram analysis. A) Time diagram from 2004 to 2023; B) Keywords with the strongest citation bursts; C) Impact flow diagram from 2018 to 2023.

Keywords from 2004 to 2023 were clustered and a time graph was generated (Supplementary Fig.5A), with nodes representing keywords and the color representing the average publication year of each node. The size of nodes was proportional to the explosion of keyword co-occurrence. The cluster name is marked on the right side of the time plot. The three primary keywords in the time diagram is neural tube defect, folic acid and red blood cell folate. From the onset, folic acid was closely related to NTDs and has received high attention. Red blood cell folate has attracted attention and become a research focus since 2015. The red line segment in the keyword emergence map indicates the duration of explosive keywords. As shown in Supplementary Fig.5B, the strongest keywords and emergence degrees were as follows: risk factor (4.26), prevention (4.23), red cell folate (4.5), hyperhomocysteinemia (4.39), prevalence (4.22), red blood cell folate (4.89). The development and trend evolution of red blood cell folic acid and pregnancy association research can be roughly defined by the time plot and emergence analysis, which can be used as a reference for predicting future research trends. The keyword co-occurrence (2018-2023) network was generated by CiteSpace software and the annual network information was input into the alluvial generator program one by one for screening and color layout to form the impact flow diagram, as shown in Supplementary Fig.5C. The width and length of impact streamlines that could help understand the occurrence intensity and influence time of keywords and the impact streamlines, such as red blood cell folate and NTDs, were wide and long and there was a close association.

### Literature co-citation analysis:

**Supplementary Fig.6:**
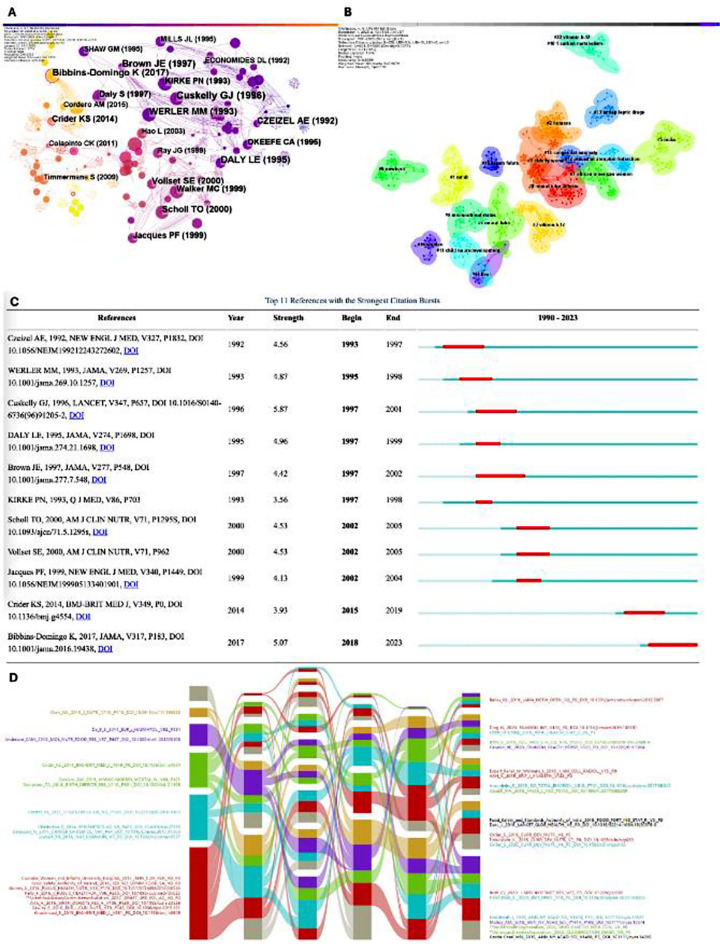
Literature co-citation analysis. Literature co-citation network and cluster analysis. A) Literature co-citation network from 1990 to 2023; B) Literature co-cited cluster network from 1990 to 2023; C) Top 11 references with the strongest citation bursts; D) Co-cited references impact flow diagram from 2018 to 2023.

The co-citation analysis of literature reflects the focus of academic research. The network formed by the co-citation analysis of red blood cell folic acid and pregnancy-related literature is shown in Supplementary Fig.6A, where the node connection represents that the two literatures have been jointly cited and the node size represents the citation frequency. All studies were of high quality and published in high-impact journals. The first key study by Cuskelly GJ et al.^11^ published in Lancet in 1996 (12) found that folic acid intake from natural foods is relatively ineffective in increasing folic acid status. The second key study by Brown JE et al.^12^ published in JAMA in 1997 (10) found that the supplementation of 400ug of folic acid per day in the diet of women of childbearing age effectively raises erythrocyte folate to levels associated with a low risk of folate-reactive neural tube defects (NTDs). The third key study by US Preventive Services Task Force and Bibbins-Domingo K et al.^13^ published in JAMA in 2017 (9) recommended that all women who plan to become pregnant or are able to become pregnant take a daily supplement containing 0.4 to 0.8 mg of folic acid. The fourth key study by Daly Le et al.^14^ published in JAMA in 1995 (9) reported that the current guidelines for the prevention of NTDs require increased folic acid intake of 0.4 mg per day. This would result in a 48% reduction in NTDs, which may be near optimal. The fifth key study by Werler Mm et al.^15^ published in JAMA in 1993 (9) showed that daily periconceptional intake of 0.4 mg of folic acid reduces the risk of occurrent NTDs by approximately 60%.

Cited studies, published from 1991 to 2023, were analyzed and 19 clusters with high significance and high confidence were formed (Q=0.9099, S=0.9679),^10^ as shown in Supplementary Fig.6B. The relevant information of the first 10 clusters is shown in Table-III. Several keywords with the highest frequency of each cluster can summarize the main direction of the cluster research and the research focus in different periods. Studies on the association between erythrocyte folate level and pregnancy based on clustering results mainly included the following aspects: (1) #7, #2 and #1 reflect the early research focus: In different regions of the population, different levels of folic acid have different effects on pregnancy outcomes, such as NTDs, placental abruption, gestational hypertension, preterm birth, spontaneous abortion and birth weight; studies have found that antiepileptic drugs can affect folic acid levels; (2) #8, #0 and #5 reflect the frontiers of research in this field: The main research focuses on the minimum requirement of folic acid in red blood cells to avoid birth defects and points out that this requirement is difficult to meet only through dietary folic acid and must be met through food fortification or folic acid supplement; Vitamin- B12 is directly involved in folic acid metabolism and its deficiency will lead to functional folic acid deficiency and damage to red blood cells.^16^

**Table-III:** Top 10 co-cited references clusters analysis of red blood cell folic acid and pregnancy.

Cluster ID	Cluster name	size	silhouette	Mean (year)	Top 5 terms (LSI)	Cited reference (Freq)
#0	Neural tube defects (NTDs)	57	0.976	2014	NTDs; dietary folate; serum folate; folic acid supplements; food fortification	Bibbins-Domingo K,2017, JAMA,V317,P183,DOI 10.1001/jama.2016.19438(9)
#1	Elderly women	57	0.953	1998	folate status; elderly women; folate catabolites; folate depletion; folate repletion	Berry RJ,1999, NEW ENGL J MED,V341,P1485,DOI 10.1056/NEJM199911113412001(4)
#2	Humans	54	0.954	1996	Placental abruption; pregnancy-induced hypertension; preterm delivery; spontaneous abortion; birth weight	Cuskelly GJ,1996, LANCET,V347,P657,DOI 10.1016/S0140-6736(96)91205-2(12)
#3	Vitamin- b12	53	0.958	2000	nutritional status; birth outcomes; ethnic minorities; methylmalonic acid; birth weight	Scholl TO,2000, AM J CLIN NUTR,V71,P1295S,DOI 10.1093/ajcn/71.5.1295s(8)
#4	MTHFR	49	0.977	2009	Mediterranean diet; dietary folate; red blood cell folate; smoke exposure; Vitamin- b-12	Colapinto CK,2011, CAN MED ASSOC J,V183,PE100,DOI 10.1503/cmaj.100568(5)
#5	India	47	0.982	2018	Vitamin- b12; Vitamin- b ; Vitamin- b12	Chen MY,2019, AM J CLIN NUTR,V109,P1452,DOI 10.1093/ajcn/nqz027(4)
#6	neural tube	44	0.973	2004	Congenital abnormalities; structural birth; neural tube; periconceptional care; preterm birth	Ray JG,2002, LANCET,V360,P2047,DOI 10.1016/S0140-6736(02)11994-510.1016/S0140-6736(02)11994-5(4)
#7	African American women	41	0.978	1993	African American women; biochemical variables; antiepileptic drugs; neural tube defect	Czeizel AE,1992, NEW ENGL J MED,V327,P1832,DOI 10.1056/NEJM199212243272602(8)
#8	Newborn	35	1	2010	Average requirement; dietary reference value; health outcomes; blood transfusion; red blood cell	Capra L,2013, ITAL J PEDIATR,V39,P0,DOI 10.1186/1824-7288-39-7(3)
#9	Micronutrient status	34	0.882	2004	Micronutrient status; assessment methods; maternal anemia; methylmalonic acid; Vitamin- b12	Hao L,2003,J NUTR,V133,P3630,DOI 10.1093/jn/133.11.3630(5)

### Research hotspot analysis based on burst literature:

Emergent literature refers to the literature with a significant increase in the number of citations at a certain stage, indicating the research hotspot in this field.^17^ As shown in Supplementary Fig.6C, the two articles published earlier in the burst literature were both studies on the association between folic acid and NTDs during pregnancy, which laid the foundation for the study on the association between red blood cell folic acid levels and pregnancy. The two most recent papers with a longer duration of outburst are Crider KS, 2014 point^18^ that used Bayesian model to study the erythrocyte folate level in population prevention of NTDs and a study by Bibbins-Domingo K et al., 2017,^13^ “Folic Acid Supplementation for the Prevention of NTDs: US Preventive Services Task Force Recommendation Statement (Recommendation for folic acid supplementation for the Prevention of neural tube defects)”.

### Impact analysis of co-cited literature on the association between erythrocyte folic acid and pregnancy based on impact flow diagram:

Through the literature co-citation network generated by CiteSpace software, the network information from 2018 to 2023 was input one by one into the alluvial generator program. After specific screening and color layout, the impact flow diagram of the representative literature on the association between folic acid in red blood cells and pregnancy was obtained, as shown in Supplementary Fig.6D. A study by Bailey RL,2019 pointed out that many pregnant women were at risk of excessive consumption of folic acid and suggested that improved dietary guidance to help pregnant women meet but not exceed dietary recommendations is warranted.^19^ A study by Egorova O,2020 suggests that high maternal serum folate status during early pregnancy may be associated with the occurrence of autism spectrum disorder in offspring.^20^

A study by Finkelstein JL,2020 conducted a population-based biomarker survey of anemia and Vitamin- B12 and folate status in women of reproductive age as part of a periconceptional surveillance program.^21^ It directly informed a randomized trial for anemia and birth defects prevention in Southern India. The study was conducted as part of a population-based WRA survey on anemia, Vitamin- B12 and folate status as part of a perinatal surveillance program and directly to inform a randomized trial on anemia and birth defect prevention in southern India.

## DISCUSSION

This study employed scientometrics to analyze trends in the association between red blood cell folate and pregnancy outcomes, providing valuable insights into personalized folate supplementation strategies. Our analysis identified key research trends and clusters, highlighting the increasing importance of folate supplementation in preventing neural tube defects (NTDs), as well as the growing emphasis on personalized care based on red blood cell folate levels. The findings reinforce the current clinical recommendations for folic acid supplementation, which suggest that all women of reproductive age should begin supplementation at least one month before conception and continue through the first trimester to reduce the risk of NTDs.^22^ This aligns with global health guidelines, particularly those from the World Health Organization (WHO), which emphasize the periconceptional period for folate supplementation.^23^ Our results also underline the need for personalized supplementation. Specifically, women with low red blood cell folate levels, especially those at higher risk of NTDs or folate deficiency, may benefit from higher doses of folic acid, such as 4 mg per day, instead of the standard 0.4 mg.^20^,^21^ A notable trend identified in our study is the increasing recognition of erythrocyte folate as a more accurate and reliable marker for assessing folate status over serum folate levels.^23^ This finding supports the integration of red blood cell folate testing into routine prenatal care, which would allow clinicians to better monitor folate status and adjust supplementation accordingly. This shift towards personalized folate supplementation has the potential to improve maternal and fetal health outcomes by ensuring that folate intake meets the individual needs of women, particularly those with chronic folate deficiency.^24^ Additionally, public health initiatives, such as folic acid fortification programs, have been successful in improving folate status globally, particularly in countries like the United States and Canada.^25^ These programs have contributed to the increasing volume of research on folate supplementation. However, even in regions with successful fortification programs, personalized care remains critical for addressing the needs of high-risk individuals, such as women with a history of NTDs or those with low folate levels despite fortification.^26^ The surge in publications since 2021 can be attributed to multiple factors, including the global emphasis on folate supplementation, advancements in research methodologies, public health campaigns, and the impact of the COVID-19 pandemic.^27^,^28^ These factors have highlighted the importance of folate in maternal health, driving further research in this area. The study also identified key authors, institutions, countries, and journals, providing a roadmap for future research. Importantly, the findings suggest that the integration of red blood cell folate testing into clinical practice could significantly enhance the prevention and management of folate deficiency. This approach could lead to more effective and personalized folate supplementation strategies, ensuring optimal folate levels for maternal and fetal health. In conclusion, this study underscores the importance of personalized folate supplementation and the need to adapt supplementation strategies to individual folate status, particularly in high-risk populations. These findings have important implications for clinical practice and public health strategies, with the potential to improve maternal and neonatal outcomes worldwide, especially in resource-limited settings.

### Biological characteristics of folic acid:

Folic acid, or pteroylglutamate, is a Vitamin- that plays an essential role in the embryogenesis of the nervous system.^30^ Folic acid participates in various metabolic reactions and is a component of the human essential amino acid (methionine), mediating the methylation of DNA.^31^ As the human body lacks the enzyme to synthesize folic acid, exhausted folic acid reserves will ultimately lead to an increased rate of double-strand DNA, triggering apoptosis.^32^

### Red Blood Cell Folic Acid:

Although folic acid is primarily stored in the liver, the nutritional status of folic acid can be assessed by measuring folic acid in urine, serum, plasma, or red blood cells.^33^ Erythrocyte folic acid level is a specific marker of clinical folic acid deficiency/deficiency.^34^ Erythrocyte folic acid level can remain stable for three to four months and the concentration of folic acid in red blood cells is 10 to 20 times higher than in serum. Therefore, this index accurately reflects the folic acid status of long-term folic acid intake.^35^ In this study, the red blood cell folic acid of pregnant women was used as a biological indicator of folic acid nutritional level. WHO defines low folic acid levels before and during the first 3 months of pregnancy as erythrocyte folic acid < 960nmol /L (400ng /mL),^34^ a threshold identified initially in the Irish population.^14^ A similar threshold was found in the Chinese population.^18^

### Role of folic acid supplementation during pregnancy:

### The preventive effect of folic acid supplementation on neural tube defects (NTDs):

Folate-sensitive birth defects include certain congenital heart diseases, urinary system infections, cleft lip and palate and limb defects, among which neural tube defects (NTDs) are the most prominent. Under normal fetal development, the embryonic neural tube begins to close on the 21st day after conception (equivalent to the 35th day after the last menstruation) and completes its closure 28 days after conception (equivalent to the 42 day after the last menstruation).^36^ Insufficient folate levels during this period may impact the fetal neural tube closure, resulting in NTDs.^37^ NTDs are one of the most common congenital malformations in the United States, with 3.9 cases of spina bifida, 2.5 cases of anencephaly and one case of encephalocele per 10,000 live births in the United States.^38^ Folic acid supplementation in the perinatal period has been found to reduce the risk of NTDs in offspring.^39^,^40^ Two high-quality studies have shown that oral administration of folic acid in women of childbearing age can significantly reduce the occurrence of NDTs, laying a foundation for subsequent studies and the current guidelines for folic acid supplementation.^39^,^41^

### Effects of folic acid supplementation on other pregnancy complications, complications and pregnancy outcomes:

Folic acid is an essential coenzyme in the process of cellular DNA synthesis. Folic acid deficiency affects DNA synthesis of juvenile erythrocytes, impacting their maturation and division and leading to the development of megaloblastic anemia.^42^ Studies have found that folic acid supplementation can reduce the incidence of anemia by 27.9%.^43^ Folic acid is also an important coenzyme in the demethylation of methamphetamine to cysteine and its content is negatively correlated with the level of homocysteine (Hcy).^44^ Hcy can affect fetal growth and development through the placenta, induce placental endothelial cell damage and even cause preeclampsia.^45^ A meta-analysis comparing prevalence before and after mandatory folic acid fortification in several countries, including Argentina, Brazil, Chile and the United States, showed significant reductions in non-syndromic cleft lip and palate^46^ and congenital heart disease.^47^ Folic acid supplementation was also associated with a 70% reduction in the risk of preterm birth at 20-28 weeks’ gestation and a 50% reduction in the risk of preterm birth at 28-32 weeks’ gestation.^48^

### Effects of folic acid on progeny:

Extensive evidence shows that pre- and post-pregnancy folic acid supplementation supports neurological and neurobehavioral development and enhances social, cognitive and language function.^49^,^50^ Studies show that folic acid supplementation during pregnancy can prevent maternal depression and cancer during the perinatal period and lower the incidence of cancer, heart disease and neurodevelopmental disorders in offspring.^7^,^51^-^55^

### Folic acid supplementation:

Public health guidelines on reducing the incidence of NTDs encourage women of childbearing age to supplement their normal diet with 0.4mg of folic acid per day,^56^ a level of intake based on clinical intervention trials^39^,^41^ and further confirmed in a population-based intervention study in China.^57^ In June 2022, the Society of Obstetricians and Gynecologists of Canada published the “Folic Acid and MultiVitamin- Guidelines for the Prevention of Congenital Abnormalities of Folic acid Sensitivity”. The guidelines emphasized personalized supplementation of folic acid to regulate and guide the scientific use of folic acid, ensure the quality and safety of health care and improve maternal health and quality of life.^58^ Based on the guidelines, women at low risk of developing NTD are recommended oral supplementation of 0.4mg of folic acid per day for at least two to three months before pregnancy, throughout pregnancy and postpartum four to six weeks or throughout lactation. Women at moderate risk of NTD (a history of abnormal folic acid sensitivity or an increased risk due to medication, surgery and/or lifestyle factors) are recommended folic acid supplementation of 1.0mg per day from pre-pregnancy to 12 weeks of gestation and a standard low-dose regimen after 12 weeks of gestation. For women at high risk for NTD (history of NTD), the supplementation regimen includes a total oral dose of 4mg of folic acid per day before pregnancy or, alternatively, a supplementation with oral multivitamin- containing folic acid (0.4 to 1.0 mg) per day for the first four to six weeks of the first three months of pregnancy, followed by the fasting serum folic acid level testing. Women with the serum folic acid levels of > 28nmol /L continue to supplement 0.4 to 1.0 mg of folic acid daily until 12 weeks of pregnancy. Women with serum folic acid levels < 28nmol /L are supplemented with daily dose that exceeds 1.0 mg. A standard low-dose regimen is resumed after 12 weeks of gestation.^58^ By reiterating the review process, the US Preventive Services Task Force found sufficient evidence that folic acid supplementation at normal doses is not associated with serious harm; while concluding with a high degree of certainty that the net benefit of folic acid supplementation is substantial for those who plan or may become pregnant.^59^

### Limitations:

One major limitation of scientometric research is the reliance on citation-based indicators, which can lead to citation bias. Citation bias occurs when studies with positive results are more likely to be cited, while those with negative or inconclusive findings may be underrepresented. In the context of folate research, this could result in an overemphasis on studies reporting positive outcomes, such as the effectiveness of folate supplementation in preventing neural tube defects (NTDs), while studies with less definitive or null results may be overlooked. Another limitation is the exclusive use of data from the Web of Science Core Collection (WOSCC), which, while comprehensive, does not capture all relevant studies. For instance, studies published in regional or non-English-language journals, such as those in Chinese, Spanish, or Arabic, may not be included in the analysis. This could limit the scope of the research, particularly for regions where folate supplementation and its impact on pregnancy are of significant concern, such as in developing countries or non-English-speaking populations. The exclusion of such studies could affect the generalizability of the findings, especially for populations with high rates of folate deficiency.^60^ Additionally, the co-citation network in this study focused primarily on the first author, which may not fully reflect the influence of all contributing authors. This limitation may underrepresent the broader collaborative impact of research teams on the field of folate supplementation.

## CONCLUSIONS

Erythrocyte folate levels are strongly associated with pregnancy outcomes, and future research should continue to focus on risk factors and preventive strategies. Based on the findings of this study, we recommend that healthcare providers use red blood cell folate as a reliable marker for assessing folate status in pregnant women. For those with red blood cell folate levels below 960 nmol/L (400 ng/mL), a higher dose of folic acid, such as 4 mg per day, should be considered, particularly for women with a history of neural tube defects (NTDs). This approach aligns with current clinical guidelines while offering a more individualized strategy that addresses deficiencies not always detected through dietary assessments alone. In resource-limited settings, where routine testing may be challenging, we recommend universal folic acid supplementation at 0.4 mg per day for all women of reproductive age, with an emphasis on preconceptional and early pregnancy supplementation to reduce the risk of NTDs. Community health workers play a crucial role in promoting folic acid supplementation, especially in underserved areas. These findings should be incorporated into both global and national clinical guidelines to ensure the wide implementation of evidence-based folate supplementation strategies, particularly in high-risk and resource-limited settings. Additionally, it is essential to monitor Vitamin B12 deficiency, as it can lead to functional folic acid deficiency, further impacting red blood cell health.

### Authors’ contributions:

**YX:** Study design, literature search, manuscript writing, revision, validation and is responsible for the integrity of the study.

**XW, DL, LS, SL and MS:** Data collection, data analysis and interpretation. Critical review.

All authors have read and approved the final manuscript.
